# Two Kinds of Mental Privacy: What Speech Decoding Does and Does Not Threaten

**DOI:** 10.1002/hast.70116

**Published:** 2026-10-28

**Authors:** Marcello Ienca

**Keywords:** mental privacy, neurotechnology ethics, brain‐computer interfaces, speech decoding, cognitive liberty, neuroscience and society

## Abstract

*In the article “Speech Restoration and Mental Surveillance as Conflicting Ethical Concerns in Neural Speech‐Decoding Research,” Narayan Sankaran and Winston Chiong distinguish speech‐decoding strategies by whether they read brain signals tied to communicative intent, softening the common equation of neural decoding with mind reading. This commentary situates their distinction within a broader conceptual map of mental privacy, borrowing Noam Chomsky's distinction between the narrow and broad senses of the language faculty. Mental privacy in the narrow sense concerns the decoding of occurrent mental states—thoughts, feelings, intentions—the domain Sankaran and Chiong address. Mental privacy in the broad sense concerns statistical inferences about traits, risks, and dispositions from brain data, which can wrong a person without there being any decoding of their mental state at all. The two senses implicate different harms, different relations to cognitive liberty, and different regulatory logics: intent‐based authorization for narrow‐sense decoding, purpose limitation and proportionality review for broad‐sense inference. Distinguishing them clarifies what current speech‐decoding research does, and does not, threaten*.

## Other Voices

In this issue of the *Hastings Center Report*, Narayan Sankaran and Winston Chiong open their article with a genuine bioethical dilemma. Speech brain‐computer interfaces (BCIs) can restore fluent communication to people who have lost the ability to speak, yet the same underlying neurotechnology feeds a public fear that machines are learning to “read minds.” Rather than treat this as an intractable clash of values, the article, “Speech Restoration and Mental Surveillance as Conflicting Ethical Concerns in Neural Speech‐Decoding Research,” looks “upstream,” to the neuroscience itself, for a possible solution.^1^ The authors distinguish decoding strategies by the location in the neurolinguistic hierarchy where they intervene: low‐level articulatory decoding, which reads motor‐cortex signals tightly coupled to a deliberate attempt to speak, and high‐level semantic decoding, which reads signals in the distributed semantic network that are active whether or not a person means to communicate. Because articulatory signals are, in Sankaran and Chiong's words, “characteristic of willful and volitional attempts to vocalize,” decoding them poses a comparatively modest surveillance risk; because semantic signals are “abstract” and “amodal,” generalizing across communicative and noncommunicative cognition alike, decoding them poses a much greater one.^2^ The authors’ proposed remedy is not to ban semantic decoding or to bless articulatory decoding uncritically but to make communicative intent itself an object of neuroscientific, conceptual, and user‐experience research—and a question that scientists, journalists, and regulators should learn to ask of every new decoding result.

This is a genuinely useful move, and it deserves to be built on rather than merely praised. My aim here is to locate Sankaran and Chiong's motor‐semantic distinction within a wider conceptual map of what we are actually worried about when we worry about “mental privacy”—a term whose folk currency, incidentally, considerably predates its philosophical career. In a 1990 Star Trek novel, Captain Kirk is talked out of ordering a telepathic search of his ship's visitors because “the right to mental privacy is an inalienable right of all Federation citizens and shall not be abrogated without due process of law.”^3^ That a mass‐market science‐fiction novel could invoke mental privacy as a settled constitutional value decades before neuroethicists began formulating it as one tells us something: the intuition is old and robust, but the concept behind it is not unitary. Sankaran and Chiong's paper, read closely, is really about only one of its two constituent senses.

## A Chomskian Distinction

Noam Chomsky and colleagues once proposed that the faculty of language be understood at two levels of resolution: a broad sense, encompassing everything the language system shares with sensory‐motor and conceptual‐intentional systems found elsewhere in cognition, and a narrow sense, the comparatively small computational core—for them, recursion—that may be unique to language and to humans.^4^ I want to borrow the logic of that distinction, not its content, to do analogous work for mental privacy.

Mental privacy in the *broad* sense, let us say, concerns the possibility of establishing statistical correlations between data—behavioral, physiological, or neural—and features of the brain or mind: identifying biomarkers associated with a particular cognitive style, a personality trait, a risk of psychiatric or neurological illness, a level of fatigue or attentional engagement. Consumer electroencephalograph (EEG) wearables inferring “focus,” resting‐state neuroimaging classifiers flagging disease risk, and workplace neuromonitoring for vigilance all belong here.^5^ Jennifer Chandler has recently pursued a version of this point in an earlier installment in this Neuroscience & Society series, examining the ethico‐legal questions raised when brain data are used to infer traits, states, or risks about a person when there is no decoding of any mental content from them.^6^ Crucially, broad‐sense mental privacy can be threatened without any decoding of any particular mental state. A biomarker for depression risk derived from spectral EEG features tells a third party something intimate about a person even though nothing was “read out” of that person's mind at the moment of testing—no thought, feeling, or intention was accessed, only a statistical propensity.

Mental privacy in the *narrow* sense, by contrast, concerns specifically the decoding of mental states: moving from brain data to information about what someone is currently thinking, feeling, intending, remembering, or perceiving. This is the sense mobilized by every one of the AI‐decoding paradigms now advancing at remarkable speed: visual reconstruction, which inverts the encoding of a perceived image in the visual cortex to generate a picture of what someone is seeing or recalling; silent‐speech decoding, of the kind Sankaran and Chiong discuss, which reads articulatory intent from the motor and premotor cortices; continuous language reconstruction, which combines brain data with the statistical expectations about probable words and phrases supplied by large language models to recover the gist of inner speech from higher‐order language networks; and, more recently, so‐called mind captioning, a hybrid approach that targets neither sensory features nor linguistic representations but the structured semantic relations—who did what to whom—that underlie a scene, generating a text description of mental content that was never spoken, written, or explicitly imagined in words at all.^7^

Whatever else divides these paradigms technically, they share the narrow‐sense structure: an attempt, successful or not, to access the content of an occurrent mental episode. And all four share a further feature worth naming, because it is routinely lost in headlines about machines that “read minds”: every one of them is mediated by a model. In technical terms, they are model dependent. None extracts a thought the way a hand might extract an object from a box. Each first learns, from a large corpus of paired brain‐and‐behavior data, how mental content of some type tends to be encoded and then infers the most probable content given a new pattern of activity—using reasoning that is closer to translation or to the way a trained reader recovers meaning from unfamiliar handwriting than to observation. That a process is interpretive rather than a literal readout does not make it innocuous. Reading a private diary is an interpretive act too—it requires knowing the language, filling in context, supplying what is left unsaid—and it is no less a violation for that; the wrong lies in unauthorized access to content someone meant to keep to themselves, not in any fantasy of unmediated access to their mind. But it does mean that the metaphor of “reading” is covering a lot of real work here, and that work deserves to be made explicit rather than left to do its damage, or its debunking, unexamined.

## Why the Distinction Matters

The two senses are not merely extensionally different; they raise distinct normative issues because they involve different relations between the knower and the known. Narrow‐sense decoding, even though it is never literal mind reading—no neural signal is a self‐interpreting message, and every decoder is a model‐mediated hypothesis about meaning, not a transparent window onto it—nonetheless aims at, and sometimes achieves, something like direct epistemic access to a specific state a person is experiencing right now. Michael Lynch has argued that this kind of access threatens something close to the structural asymmetry that makes a person a subject rather than an open book to others. Imagine, he writes, that someone could telepathically read all your conscious and unconscious thoughts and feelings: “your existence as a distinct person would begin to shrink.”^8^ Narrow‐sense violations are objectionable, in other words, largely independently of what is subsequently done with what was accessed.

Broad‐sense inference is objectionable for a different reason. Its harm is not access to an occurrent state but the construction, from otherwise innocuous correlational patterns, of a profile that can be used to sort, predict, price, exclude, or discriminate against a person—the same worry we have about genomic or behavioral data more generally, only compounded by the fact that brain data are unusually rich, longitudinal, and involuntarily generated.^9^ One can be wronged in the broad sense while remaining, at the level of narrow‐sense decoding, entirely opaque: nobody need ever know what you are thinking for a biomarker classifier to have already decided something consequential about you.

## Locating Sankaran and Chiong within the Map

Once this distinction is on the table, it becomes clear that Sankaran and Chiong's motor‐semantic taxonomy is an internal taxonomy of narrow‐sense mental privacy. Both articulatory and semantic decoding aim at the content of an occurrent state—an intended utterance versus a broader thought—and the authors’ contribution is to show that, even within the narrow sense, communicative intent differentiates degrees of privacy risk. That is an important and, to my knowledge, novel refinement, and it usefully softens the crude equation of “neural decoding” with “mind reading” that dominates journalistic coverage. But it leaves broad‐sense mental privacy essentially untouched. A speech BCI trained to decode articulatory intent for restorative purposes could, incidentally and without decoding a single sentence, also serve as a rich longitudinal biomarker source—for neurodegenerative progression, medication response, or cognitive decline—precisely because motor‐cortex signals are informative about far more than the specific words a user means to say. Communicative intent is the right question to ask about a given decoded output; it is not obviously the right question to ask about a data stream's downstream statistical utility.

This is also, I think, the cleanest way to see why concerns about mental privacy do not simply collapse into Nita Farahany's account of cognitive liberty, with which they are often conflated.^10^ Cognitive liberty is best understood as a right of self‐determination over one's mental life—a right against manipulation, coercion, and unauthorized alteration of one's cognitive processes, exercised primarily against interference. Mental privacy, in both its senses, is a right against unauthorized access or use—exercised against exposure. The two interests overlap in practice, since unauthorized access often enables unauthorized influence, but they are not the same right, and the narrow‐broad distinction shows why a single unified “neurorights” framework, however useful for advocacy, may be doing conceptual work that actually requires two different regulatory instruments: something like informed, purpose‐bound consent for narrow‐sense decoding and something more like data‐protection‐style purpose limitation, necessity, and proportionality review for broad‐sense inference.^11^

That last point bears on how we should think about privacy as what philosophers of law call a “relative,” rather than “absolute,” right—one that must be balanced against competing interests rather than honored unconditionally.^12^ Sankaran and Chiong's proposal is, properly understood, a relativizing move for the narrow sense: because articulatory decoding is more reliably tied to communicative intent, it can be authorized more readily than semantic decoding, which requires additional safeguards precisely because its ties to intent are looser. The same relativizing logic applies to the broad sense, but the balancing test looks different. There, permissibility should turn less on the presence of intent—there may be none to speak of—and more on whether use is bound to the purpose for which the data were generated, whether inference is necessary and proportionate to that purpose, and whether independent oversight exists to prevent repurposing: the kind of context‐sensitive, purpose‐limited authorization regime that data‐protection law, for all its gaps regarding brain data, was built to provide.^13^

## A Milestone Worth Insisting On

None of this diminishes what Sankaran and Chiong have accomplished. Their insistence that ethical scrutiny of speech decoding should track the neuroscience—where in the processing hierarchy a signal originates and how tightly it is coupled to a deliberate communicative act—is exactly the kind of upstream, scientifically literate ethics that this field needs more of, and their three questions for journalists are a genuine public service. What I am proposing is that we need not choose between an ethics anchored to communicative intent and a broader ethics of statistical inference; we should build both—because they answer different questions about what a brain‐reading technology is doing to a person.

As neurotechnology moves from single‐purpose motor decoders toward the kind of AI‐mediated, foundation‐model‐driven decoding now emerging in laboratories—where the same architecture that reconstructs a sentence from cortical activity can, with a different objective function, just as easily be repurposed to infer a trait—the narrow‐broad distinction will become only more, not less, load bearing. Getting it right now, while the relevant devices are still mostly confined to clinical trials rather than sitting on store shelves, is the difference between designing safeguards for the technology we are actually building and designing them for the one that made headlines.

## Acknowledgments

This study has been funded by the German Federal Ministry of Research, Technology and Space (BMFTR), under grant number 01GP2506A (funding the NeuroAI ELSA Observatorium project), and the German Research Foundation (DFG) under grant number 533801653 (funding the TransforM—Munich Center for Transformative Technologies and Societal Change project).

This paper is part of a series, Neuroscience and Society, whose development by The Hastings Center for Bioethics is funded by the Dana Foundation.
